# The Herbert Hospital, Woolwich—A Grave Scandal

**Published:** 1900-01-13

**Authors:** 


### EDITOR'S LETTER-BOX.

[Our Correspondents are reminded that prolixity is a great bar to publication, and that brevity of style and conciseness of statement greatly facilitate early insertion.]

### THE HERBERT HOSPITAL, WOOLWICH—A GRAVE SCANDAL.

A VALUED correspondent writes: I have been hearing such a deplorable account of the Government Hospital at Woolwich. It appears there are six sisters—no nurses, and the former are only allowed to take temperature and give medicines. The rest of the work is done by orderlies. The experienced ones having gone to Africa, they only consist of boys knowing nothing at all, and absolutely incompetent. There are a great many cases of enteric fever from South Africa, and as the rules of the hospital forbid the sisters to remain in the wards they are powerless. One sister is on night duty, but there is no matron or head. The cases want watching, and are terribly neglected. Surely it is work for women, not careless boys? I do not know if it is possible to do anything. It seems so hard to feel everything is not done for the poor fellows who are suffering for us and their country. I felt I must tell you these facts. We are doing so much for those away, it is hard to feel they are not properly looked after at home.

*** The above complaint is one which we think deserves the most careful consideration. Of course, we know what is the answer which might and probably would be given to it in official quarters, namely, that the whole organisation of our army, including the arrangements for the sick, is one which has been worked out with the object of being available in war, of being fitted for service in the front, and of being able to continue its operations without any change amid the varied circumstances of the battlefield. An apt illustration is in evidence on every side. When we see a heavy service waggon lumbering through the streets we are apt to say what a clumsy, rough-and-ready contrivance—surely, our civilisation could turn out something better than that! The answer is, however, that the waggon must be of such construction that it will bear not only the friction of the ordinary road, but be able to go anywhere, and if it should break down be capable of being mended by rough-and-ready methods and by very rough-and-ready artificers. The same with our military hospitals; they are organised for service, and hospitals so organised as to be capable of taking up their position in the field must, it is said, be nursed by orderlies; and although "sisters" are allowed to cast their civilising influence upon them, and are permitted to teach the orderlies so much of the art of nursing as they can, the hospital must be so arranged as to be capable of working smoothly, and without derangement of its accustomed method, even if no sisters are present, and even if bomb shells are flying all around. That, we take it, is the excuse for the fact that our great military hospitals are without nursing, as the term is understood in civil hospitals. Two further reasons, however, may be mentioned. One is that the sort of cases with which a large proportion of the beds are filled in time of peace are such as, after all, would have to be dealt with by male rather than by female nurses, and the other is that in ordinary times, owing to the fact that if a man is off duty he must be in hospital, a considerable proportion of the cases in the wards are not suffering from such serious disease as to require much nursing of any sort. Such are, no doubt, valid excuses in times of peace, when there is never any pressure and when the hospitals are properly regarded as places in which the orderlies may be trained for the work which in time of war will fall upon them. But in times of war things are different, and we think that all who are able to cut themselves adrift from red tape traditions will agree that it is a grave scandal that while, as is the case at present, we are entirely upsetting our usual arrangements by substituting civilian for military medical officers, and by providing skilled nursing for our hospitals at the Cape, the hospitals at home, denuded as they are of all the better class of orderlies and filled, as they soon will be, by severe cases from the front, should remain tied down by the old regulations, according to which the nursing is handed over to those who are, from the circumstances of the case, necessarily the least skilled of an unskilled class. Between a military hospital on its ordinary footing and one filled with serious cases there is no possible resemblance, and when we remember the care which in our civil hospitals is lavished on patients suffering from enteric fever, we cannot without indignation contemplate the fact that when our soldiers are stricken down by this disease they are handed over to the tender mercies of such amateur nurses as our correspondent speaks of. What has been done in the direction of sending out consulting surgeons, and civilian surgeons, and nurses to South Africa, all of which has met with marked approval on every hand, has been done with the object of giving to our soldiers in their hospitals the same advantages as are enjoyed by civilians in theirs, and it is absurd that when our sick are landed here they should be placed under a system devised for quite different conditions. We have no doubt many excuses will be made, but the nursing of enteric fever is serious work on which life often depends, and we hold it to be a scandal deserving the strongest possible condemnation and demanding immediate reform, that while in the great fever hospital at the bottom of the Hill at Woolwich all sorts of people, including the very sweepings of the streets and those who from their modes of life have least deserved our sympathy, are being nursed back into health by aid of everything that money can provide, our soldiers who for us and for their country are suffering from wounds and are laid low with fever in that other hospital at Woolwich should, in the name of red-tape and military discipline, be deprived of that kind of help in sickness which for all other classes of the community is regarded as essential.—ED. *T.H.*

